# Prevalence of depression and its association with quality of life in patients after pacemaker implantation during the COVID-19 pandemic: A network analysis

**DOI:** 10.3389/fpsyt.2023.1084792

**Published:** 2023-03-16

**Authors:** Yun Lin, Hong Cai, Hong-Hong Liu, Xue-Jian Su, Chen-Yu Zhou, Jing Li, Yi-Lang Tang, Todd Jackson, Yu-Tao Xiang

**Affiliations:** ^1^Department of Cardiology, Beijing Anzhen Hospital, Capital Medical University, Beijing, China; ^2^Unit of Psychiatry, Department of Public Health and Medicinal Administration, Institute of Translational Medicine, Faculty of Health Sciences, University of Macau, Macao, Macao SAR, China; ^3^Centre for Cognitive and Brain Sciences, University of Macau, Macao, Macao SAR, China; ^4^The National Clinical Research Center for Mental Disorders and Beijing Key Laboratory of Mental Disorders, Beijing Anding Hospital and the Advanced Innovation Center for Human Brain Protection, Capital Medical University, Beijing, China; ^5^Department of Psychiatry and Behavioral Sciences, Emory University, Atlanta, GA, United States; ^6^Atlanta VA Medical Center, Atlanta, GA, United States; ^7^Department of Psychology, University of Macau, Macao, Macao SAR, China

**Keywords:** pacemaker implantation, depression, quality of life, network analysis, COVID-19

## Abstract

**Background:**

This study was designed to investigate the prevalence and predictors of depression in patients after pacemaker implantation during the COVID-19 pandemic in addition to identifying specific depressive symptoms associated with quality of life (QOL) using network analysis (NA).

**Methods:**

This cross-sectional, observational study was conducted in China between July 1, 2021, and May 17, 2022. Descriptive analysis was used to calculate depression prevalence. Univariate analyses were used to compare differences in demographic and clinical characteristics between depressed and non-depressed patients following pacemaker implantation. Binary logistic regression analysis was used to assess factors independently associated with depression. Network analysis “expected influence,” and flow function indexes were used to identify symptoms central to the depression network of the sample and depressive symptoms that were directly associated with QOL, respectively. Network stability was examined using a case-dropping bootstrap procedure.

**Results:**

In total, 206 patients implanted with a pacemaker met the study entry criteria and completed the assessment. The overall prevalence of depression (PHQ-9 total score ≥ 5) was 39.92% [95% confidence interval (CI) = 29.37−42.47%]. A binary logistic regression analysis revealed that patients with depression were more likely to report a poor health status (*p* = 0.031), severe anxiety symptoms (*p* < 0.001), and fatigue (*p* < 0.001). In the network model for depression, “Sad mood,” “Poor Energy,” and “Guilt” were the most influential symptoms. “Fatigue” had the strongest negative association with QOL, followed by “Sad mood” and “Appetite”.

**Conclusion:**

Depression is common among patients having undergone pacemaker implantation during the COVID-19 pandemic. Anxiety, central symptoms of depression (i.e., “Sad mood”, “Poor Energy”, and “Guilt”) and depressive symptoms linked to QOL (i.e., “Sad mood”, “Appetite”, and “Fatigue”) identified in this study are promising targets for interventions and preventive measures for depression in patients who have undergone pacemaker implants.

## 1. Introduction

Due to lifestyle changes, urbanization, and accelerated population aging, heart diseases, including heart rhythm disorders, have increased in China during the past decade (1). One common heart rhythm disorder, bradyarrhythmia, refers to an abnormally slow resting heart rate, typically below 60 beats per minute. Currently, there is no oral medication to treat bradyarrhythmias and, in some cases, implanting a pacemaker is the only viable treatment option. Pacemaker implantation involves placing a small device in the chest to help the heart beat at a normal rate and rhythm (2). Many cardiovascular diseases, including atherosclerotic cardiovascular disease (ASCVD), heart failure, cardiomyopathy and valvular diseases, may lead to bradyarrhythmias during later disease stages. Therefore, the demand for pacemaker implantation has increased with rises in aging populations (3–6).

For example, an estimated 600,000 pacemakers are implanted per annum globally while another 3 million people already have pacemakers (7). Implantation rates have increased by an estimated 10−15% per year and this upward trend is expected to continue during the next decade (6). In China, total cardiac pacemaker implantations reached 100,230 cases in 2019, 99,247 cases in 2020, and 111,678 cases in 2021 (8), with elderly patients as the most rapidly growing population segment (6). While implanted devices are often life-saving for those with life-threatening arrhythmias, implantations can also be life-altering and recipients experience numerous challenges in the psychosocial adaptation process (9). After pacemaker implantation, patients may confront physical limitations (e.g., limited mobility), financial strain (e.g., medical costs), psychosocial disruptions such as lowered quality of life (QOL), and existential concerns related to their illness and living with a pacemaker (10).

Associations between heart rhythm disorders and depressive symptoms (depression thereafter) are well-documented and assumed to be bi-directional in nature (11, 12). On one hand, patients with heart rhythm disorders often have a higher risk of developing depression compared to those without heart rhythm disorders. For instance, one study found that the prevalence of depression after pacemaker implantation was 41% among patients in Turkey (13). In part, biological changes associated with heart rhythm disorders have been linked to an increased risk for depression (12). Meta-analyses have found increased C-reactive protein (CRP) and interleukin-6 (IL-6) in CVD patients, both of which are associated with higher risk of depression (14). Conversely, depression may also precipitate or exacerbate heart rhythm disorders as well as associated risk factors (e.g., hypertension, insulin resistance, diabetes, and treatment adherence) (15, 16).

Aside from depression, post-implantation QOL is another important facet of psychosocial adjustment warranting consideration in these patients (17–20). After pacemaker implantation, patients often experience psychological consequences (e.g., depression, anxiety, and fatigue) related to change of lifestyle, limitations in daily activities, and physical discomfort, that lower their QOL (19). Previous studies have found association between higher overall depression severity and lower QOL (17, 18), but inter-relationships between specific depressive symptoms and QOL have not been well documented in bradyarrhythmia patients after pacemaker implantation.

Traditionally, the presence of mental health problems such as depression has been determined on the basis of symptom counts on interviews or total scores and cut-off values from validated questionnaires. However, the assumption that individual symptoms are equally-weighted expressions within a single underlying disorder in traditional statistical approaches to psychopathology assessment has been questioned (21–23). For example, the reliance upon average or total scale scores fails to consider potential causal relations, progressions, and heterogeneity of individual symptoms as well as interrelations between different symptoms (22, 24). In contrast, network approaches offer novel statistical methods in which mental health problems are viewed as systems of interacting symptoms that may give rise to each other (25). Network analysis has the potential to map specific relationships among individual symptoms of a disorder/syndrome, pinpoint symptoms that link particular syndromes to other experiences such as QOL, and identify specific symptoms as plausible treatment targets (23). In network theory, central nodes are the most influential symptoms of a disorder/syndrome that can activate other symptoms. Central symptoms play a major role in causing the onset and/or maintenance of a syndrome. Network analysis may have utility in clarifying features of depression that are more critical to understanding inter-relationships between different symptom clusters in under-studied target populations including patients who have undergone pacemaker implantations.

Finally, the COVID-19 pandemic has led to further complications for health service provision in many countries including China. For example, many emergency, intensive, or intermediate care units undertook heavy additional treatment burdens and some wards in regional and tertiary hospitals were converted to COVID-19 isolation units (26). Consequently, regular medical services have been reduced. Following pacemaker implantation, patients need to attend regular follow-up clinics but reduced medical services and other strict public health measures are potential barriers that interfere with regular assessments (27, 28); uncertainty in aftercare may contribute to increased risk for depression. Furthermore, compared to rates in the general population, COVID-19 vaccine rates in patients with major medical conditions including heart diseases are typically lower in China (29–31); such trends may contribute to higher infection rates or complications among the medically vulnerable that, in turn, increase depression risk.

Based on this overview, this study had three main objectives. First, we assessed the prevalence of depression among patients who had undergone pacemaker implantation during the COVID-19 pandemic. Second, we examined participant characteristics that predicted depressed versus non-depressed status within the research sample. Third, we explored specific depressive symptoms that were most central to depression and QOL among participants.

## 2. Materials and methods

### 2.1. Sampling and sample size estimation

This was a cross-sectional, observational study conducted between July 1, 2021, and May 17, 2022 at the National Clinical Research Center for Cardiovascular Diseases in Beijing, China. Following other studies (32, 33), the WeChat-based “QuestionnaireStar” program was used to collect data. WeChat is a widely used social communication application with more than 1.2 billion active users in China. All patients who had pacemaker implantations and regularly attended their follow-up clinics for maintenance therapy during the study period were consecutively invited by a research physician to participate in this study. Patients needed to present their WeChat-based health code during the pandemic when they entered the clinic and were, presumably, WeChat users. To be eligible, patients met the following selection criteria: (1) aged 18 years or older; (2) received a pacemaker implantation; (3) able to read and understand Chinese. Those with dementia and obvious cognitive problems that interfered with comprehension were excluded.

The sample size was calculated using the formula *N* = Za^2^P (1–P)/d^2^, in which *a* = 0.05 and *Z*_*a*_ = 1.96, and the estimated acceptable margin of error for proportion d was 0.05. The prevalence of depression among older population was estimated to be 35.1% based on a previous study (34). Assuming that 10% of those invited would refuse participation in this study, a sample size of at least 249 participants would be ideal.

### 2.2. Data collection

Patients were invited to scan a Quick Response code (QR Code) linked to the introduction and invitation of this study with their smartphone prior to their clinic appointments. After providing the electronic written informed consent, they were asked to complete the online assessment using their smartphone at an outpatient clinic.

Socio-demographic data were collected using a pre-designed data collection sheet and included gender, age, body mass index (BMI, kg/m^2^), marital status (married/unmarried), education level (high school and below/college education and above), having medical insurance, current smoking, current social drinking, perceived health status and perceived economic status (poor or fair/good). Following a previous study (35), standard (no versus yes) questions related to the pacemaker implantation were asked including the following: (1) “Have you experienced chest discomfort?”; (2) “Have you been restricted by chest discomfort during physical activities?”; (3) “Have you felt discomfort in the region of the intervention (chest/groin)?”; (4) “Have you been restricted in your daily activities by fear of complications?”; (5) “Since implantation, have you felt preoccupied with your heart condition and general health?.”

### 2.3. Measurement

Severity of depressive symptoms was measured using the validated Chinese version of the nine item-Patient Health Questionnaire (PHQ-9) (36, 37). PHQ-9 items include (1) “Anhedonia”, (2) “Sad Mood”, (3) “Sleep”, (4) “Energy”, (5) “Appetite”, (6) “Guilt”, (7) “Concentration”, (8) “Motor disturbance”, and (9) “Suicidal ideation”, each of which is rated from 0 (define meaning of “0” anchor here e.g., “not at all”) to 3 (define anchor meaning). Total PHQ-9 scores range from 0 to 27; values of ≥ 5 indicate the presence of depression (36, 37) while values ≥ 10 reflect “having moderate to severe depression.” Severity of anxiety was assessed using the validated General Anxiety Disorder (GAD-7) (38, 39), with total scores ranging from 0 to 21. Severity of fatigue was assessed using a one-item fatigue numeric rating scale with anchors ranging from “0” (no fatigue) to “10” (extreme fatigue) (40). Finally, global QOL was measured with the first two items of the validated World Health Organization Quality of Life Scale Brief version (WHOQOL-BREF): “How do you assess your quality of life?” and “Are you satisfied with your current health?” (41, 42). Higher scores reflected higher QOL.

### 2.4. Ethical approval

The study protocol was approved by the Clinical Research Ethics Committee of Beijing Anzhen Hospital.

### 2.5. Statistical analysis

#### 2.5.1. Univariate and multivariate analyses

Data analyses were performed using SPSS version 25.0 (SPSS Inc., Chicago, IL, USA). Distributions of continuous variables were checked for normality using P-P plots. Mean PHQ-9 scores were calculated to estimate depression prevalence in the sample. Chi-square tests, independent samples *t*-tests, and Mann-Whitney *U*-tests were used to compare sociodemographic and disease-related variables between depression and no depression groups, as appropriate. Binary logistic regression analyses with the “enter” method were performed to examine independent correlates of depression. All variables that had significant group differences in univariate analyses were entered as independent variables, while depression was entered as the dependent variable. Independent associations of depression with QOL were examined using analysis of covariance (ANCOVA) after controlling for variables on which there were significant depression subgroup differences in univariate analyses. Significant statistical differences were set at *P* < 0.05 (two-tailed).

#### 2.5.2. Network structure

The network model was estimated using R software (43). We computed polychoric correlations of all PHQ-9 items to investigate edges of the network model. We also estimated the Graphical Gaussian Model (GGM), a popular network model, with the graphic least absolute shrinkage and selection operator (LASSO) and Extended Bayesian Information Criterion (EBIC) model using R package “*qgraph”* (44). GGM is a pairwise Markov random field (PMRF) model used for interval or ordinal data; edges are interpreted as partial correlation coefficients. The network was visualized using the “*qgraph”* package, where thicker edges represented stronger relationships between nodes. We used the centrality index, Expected Influence (EI) of nodes, to identify depressive symptoms that were more central (influential) in the network model (45). To identify particular depressive symptoms that were directly associated with QOL, the “flow” function in R package “*qgraph”* was used (46).

#### 2.5.3. Network stability

Centrality stability was examined using the correlation stability coefficient (CS-coefficient). A CS-coefficient value above 0.25 indicates that observed network model results are stable, though traditionally, CS-coefficient values above 0.5 are preferable. A bootstrapped difference test was conducted to assess the robustness of node EIs and edges. Differences were significant between two nodes or two edges if zero was not included in the 1,000-bootstrap 95% confidence interval (CI). Edge accuracy was estimated with bootstrapped 95% CIs; a narrower CI suggests a more reliable network. These procedures were conducted using the package “*bootnet”* v1.4.3 (47).

## 3. Results

### 3.1. Participant characteristics

Of 210 pacemaker implantation recipients who were invited to participate in the study, 206 met the study entry criteria and completed the assessment, for a participation rate of 98.1%. Demographic and clinical characteristics of the sample are shown in Table 1. The mean age of participants was 68.65 years [standardized deviation (SD) = 12.48 years] and 51.5% (*n* = 106) were men.

**TABLE 1:** Demographic characteristics of participants.

Variable	Total (*N* = 206)		No DEP (*N* = 132)		DEP (*N* = 74)		Univariate analyses		
	***N***	**%**	***N***	**%**	***N***	**%**	**χ^2^**	**Df**	***P***
Male gender	106	51.5	71	53.8	35	47.3	0.800	1	0.387
College education and above	65	31.6	43	32.6	22	29.7	0.178	1	0.755
Married	44	21.4	23	17.4	21	28.4	3.387	1	0.077
Having medical insurance	204	99.0	131	99.2	73	98.6	0.174	1	1.00
Perceived good economic status	23	11.2	18	13.6	5	6.8	2.263	1	0.169
Perceived good health status	45	21.8	43	32.6	2	2.7	24.785	1	**<0.001**
Nosmoking	157	76.2	100	75.8	57	77.0	0.042	1	0.866
Social drinking	45	21.8	30	22.7	15	20.3	0.168	1	0.728
Having chest discomfort	87	42.2	41	31.1	46	62.2	18.801	1	**<0.001**
Chest discomfort during physical activities	124	60.2	71	53.8	53	71.6	6.294	1	**0.017**
Discomfort in the region of the intervention (chest/groin)	71	34.5	38	28.8	33	44.6	5.245	1	**0.032**
Restricted in your daily activities due to fear complications	104	50.5	53	40.2	51	68.9	15.698	1	**<0.001**
Worry about heart condition and general health	93	45.1	49	37.1	44	59.5	9.555	1	**0.002**
	**Mean**	**SD**	**Mean**	**SD**	**Mean**	**SD**	**t/Z**	**Df**	***P***
Age (years)	68.65	12.48	67.70	12.79	70.35	11.79	−1.469	204	0.144
BMI (Kg/m^2^)	25.25	4.84	25.60	4.55	24.62	5.28	1.403	204	0.162
GAD-7 total	2.92	4.38	0.67	1.22	6.95	5.05	657.000	—*	**<0.001**
Fatigue total	2.96	2.06	2.08	1.59	4.53	1.87	1600.500	—*	**<0.001**
Global QOL	6.62	1.45	7.23	1.19	5.53	1.22	9.496	204	**<0.001**

Bolded values < 0.05; DEP, depressive symptoms; M, mean; SD, standard deviation; BMI, body mass index; GAD-7, 7-item general anxiety disorder; QOL, quality of life; *Mann-Whitney *U*-test.

### 3.2. Prevalence and correlates of depression

The overall prevalence of depression (PHQ-9 total score ≥ 5) was 39.92% [95% confidence interval (CI) = 29.37−42.47%], while the prevalence of moderate to severe depression (PHQ-9 total score ≥ 10) was 14.98% (95% CI = 10.11−19.84%). Table 1 summarizes comparisons of demographic and clinical characteristics for depressed versus non-depressed pacemaker implantation patient subgroups. The depressed subgroup reported a poorer self-assessed health status (*p* < 0.001), higher mean GAD-7 total score (*p* < 0.001), and higher mean fatigue total score (*p* < 0.001). The depressed subgroup was also more likely to report having chest discomfort (*p* < 0.001), discomfort in the intervention region (chest/groin) (*p* = 0.032), severe restrictions in daily activities due to fear of complications (*p* < 0.001) and worry about their heart condition and general health (*p* = 0.002). After controlling for other significant depression subgroup differences, the ANCOVA revealed the depressed subgroup had significantly lower QOL scores than the non-depressed subgroup did [*F*_(1_, _206)_ = 47.728, *P* < 0.001].

A binary logistic regression analysis indicated depressed patient subgroup members reported a comparatively poorer perceived health status (*p* = 0.031), more severe anxiety symptoms (*p* < 0.001) and higher levels of fatigue (*p* < 0.001). No other univariate correlates had statistically significant effects in the multivariate prediction model (Table 2).

**TABLE 2:** Independent correlates of depression in study sample.

Variable	Depression		
	***P***	**OR**	**95% CI**
Good health status	**0.031**	0.077	0.007–0.795
Having chest discomfort	0.280	2.104	0.546–8.115
Chest discomfort during physical activities	0.781	0.821	0.204–3.310
Discomfort in the region of the intervention (chest/groin)	0.066	0.247	0.055–1.099
Restricted in your daily activities due to fear of complications	1.00	0.322	0.083–1.243
GAD-7 total	**<0.001**	3.165	2.064–4.848
Fatigue total	**<0.001**	2.477	1.664–3.688

Bolded value < 0.05; CI, confidential interval; OR, odds ratio; There was collinearity between worry about heart condition and general health and GAD7 total score; therefore, only GAD-7 total score was entered in the logistic regression analysis.

### 3.3. Network structure of depressive symptoms

**FIGURE 1:**
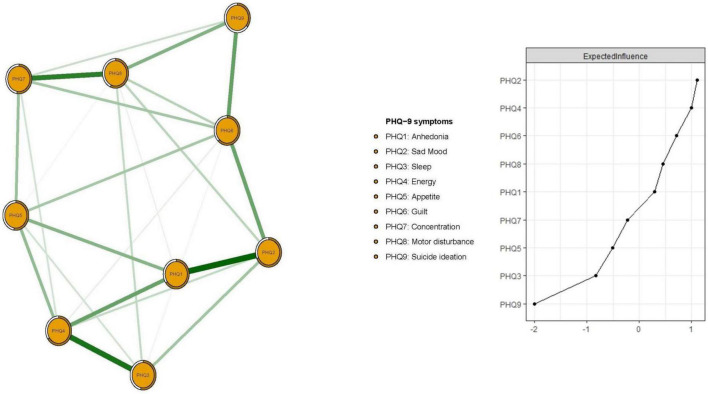
Network structure of depression in patients after pacemaker implantation.

Figure 1 presents the network structure of depressive symptoms as measured by PHQ-9 items. The predictability of items is shown as ring-shaped pie charts in Figure 1. The mean predictability was 0.566, indicating that, on average, 56.6% of the variance in each node could be accounted for by neighboring nodes in the model. The connection between nodes PHQ1 (“Anhedonia”) and PHQ2 (“Sad mood”) (average edge weight = 0.435) was the strongest positive edge, followed by edges between nodes PHQ3 (“Sleep”) and PHQ4 (“Energy”) (average edge weight = 0.390), and nodes PHQ7 (“Concentration”) and PHQ8 (“Motor disturbance”) (average edge weight = 0.360).

**FIGURE 2:**
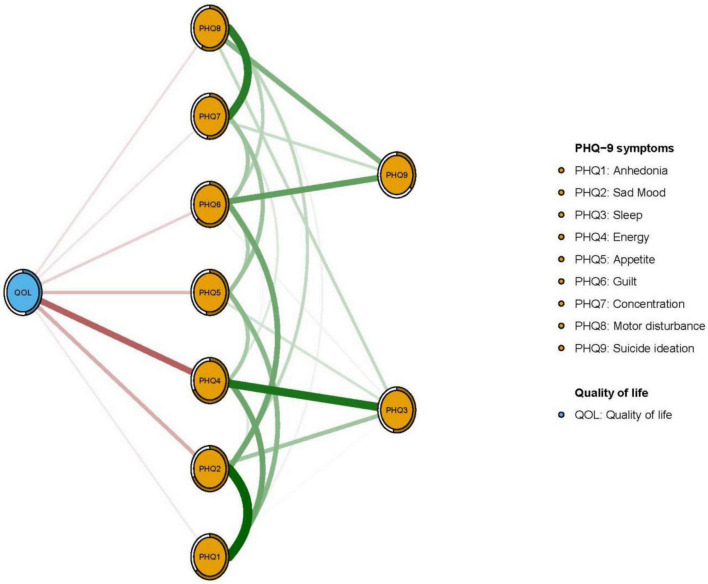
Flow network for quality of life (QOL) in study sample.

In terms of EI in the network model, the node PHQ2 (“Sad mood”) had the highest EI centrality, followed by nodes PHQ4 (“Energy”) and PHQ6 (“Guilt”) (Figure 1); together, these were the most influential symptoms for understanding depression in patients who had pacemaker implantations. In addition, PHQ4 (“Fatigue”) had the strongest negative association with QOL (average edge weight = −0.262), followed by PHQ2 (“Sad mood”) (average edge weight = −0.134) and PHQ5 (“Appetite”) (average edge weight = −0.118) (Figure 2).

**FIGURE 3:**
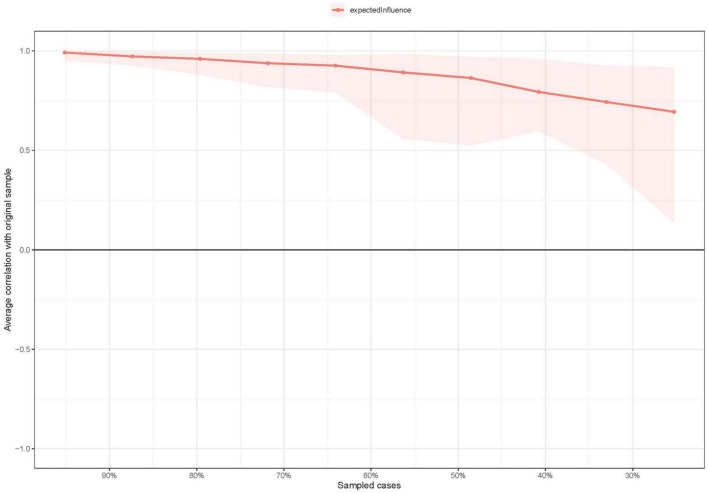
The stability of centrality index using case-dropping bootstrap.

EI centrality of the network model had moderate stability (i.e., CS-coefficient = 0.437; 95% CI: 0.359–0.515). Results of bootstrapped differences tests for edge weights showed that most comparisons between edge weights were statistically significant. As such, the network model had acceptable reliability and stability (Figure 3 and Supplementary Figure 1).

## 4. Discussion

To our knowledge, this is the first study to examine the prevalence and prediction of depression among patients who had undergone pacemaker implantation as well as key symptoms of depression and their associations with QOL using network analysis. In this study the prevalence of depression was 39.92% (95% CI: 29.37–42.47%), a rate that is similar to that reported in Turkey (36.9%) based on the modified Hamilton Depression Rating Scale (48), but much higher than rates from Iran (7.1%) based on the Beck Depression Inventory (19) and New Zealand (17.3%) as assessed with the Hospital Anxiety and Depression Scale (49). The higher prevalence of depression in this study than other studies may be due to differences in sampling methods and patient characteristics (e.g., inpatients vs. outpatients), illness stage, and depression measures. Nonetheless, chronic illness patients treated with implantation devices are confronted with considerable uncertainty that may contribute to depression (50–52).

For example, after pacemaker implantation, lower physical activity levels, obesity, high stress levels, and hypertension could contribute to depression (53). Adopting coping strategies that include focusing on activities, maintaining social support from loved ones and having adequate rest, may improve depressive symptoms. Patient support groups also provide opportunities for the exchange of information and ideas, coping options and alternative perspectives about implanted devices that may help to combat depression and/or QOL losses. Additionally, educational interventions designed to highlight the goals, functions, and positive effects of implantation devices may help to curb depression in some cases (19, 54).

Several factors including poor perceived health status, more severe anxiety and heightened fatigue emerged as the most significant, unique correlates of overall depression scores in this sample. These findings dovetail with previous findings documenting higher risk for severe depression among pacemaker implantation recipients due to more highly compromised immune systems (55), poorer health status and limited access to appropriate care during the COVID-19 pandemic (56), anxiety symptoms, increased sleep disturbances (57), fatigue, and lower socioeconomic status (52).

In the depression network model, “Sad mood” was the most central symptom, echoing previous findings among widowed older people in China (58), depressed outpatients (59), and older residents of Hong Kong (60). In late life, sad mood may result from adverse life events (i.e., severe physical diseases, implementing devices, losses of function, grief from interpersonal losses) and poor social support. Furthermore, fear of device malfunction or over-dependence on health professionals, limitations in daily activities due to pacemaker implantation, physical discomfort, and technical issues including battery depletion may trigger feelings of helplessness or sad mood in patients after pacemaker implantation (17, 51, 52, 61, 62). Moreover, in the context of the COVID-19 pandemic, restrictive public health measures including mass quarantines, facility closures, and restrictions on public transport (63–65) were adopted in many areas of China. Consequently, increased disruptions to daily life and decreased access to treatment among pacemaker implantation recipients may have contributed to exacerbations in sad mood and fatigue as well as lowered QOL.

The node “Energy” was another significant central symptom in the depression network model, consistent with previous findings reported in community-dwelling older adults (66), patients with major depressive disorder (67) and adult Hong Kong residents (60). Older adults with pacemaker implantation may experience disturbances in sleep and appetite that influence energy levels (68). Moreover, restricted outdoor exercise due to quarantines from the COVID-19 pandemic could fuel fatigue and energy depletions many patients feel (69).

Guilt also emerged as a central symptom in the depression network model in line with findings from older adult residents of Hong Kong (60). Participants in this study were typically older adults. Compared to their younger counterparts, older adults are more likely to experience chronic physical illness (e.g., hypertension, heart disease, diabetes, cancer, and stroke) (70, 71), social isolation, a lower socioeconomic status, vision deficits and cognitive impairments (72, 73), loneliness (74) and heavy healthcare burdens (75); hence, older adults may experience increases in guilt, in part, because they view themselves as a burden for families, the medical system, and society due to functional losses from aging and illness (76–78). Follow-up treatments for pacemaker implantation recipients may increase personal and financial burdens for patients’ families and the healthcare system, hence contributing to more pronounced feelings of guilt in affected patients. Particularly in the context of COVID-19, financial strain may worsen if family members were made redundant or forced to stay at home without income during the pandemic.

Fatigue, a common symptom among pacemaker implantation patients, also emerged as a central symptom linked to QOL in the network model. This observation is consistent with previous evidence implicating fatigue as a prevalent, severe symptom in heart disease patients with lower QOL (51, 61, 79). Due to reduced motivation and/or energy in performing activities of daily living and potential changes in sleep patterns, depressed patients often experience increased fatigue (80). Once again, prolonged anxiety, fear, and stress responses of pacemaker implantation patients may be even more elevated as a result of living in uncertain and unrelenting COVID-19 pandemic conditions. Over time, such reactions may contribute to high levels of fatigue and lowered QOL.

Within the flow network model, sad mood was negatively associated with QOL, consistent with evidence from another study linking negative mood states with poorer QOL among advanced heart failure patients (81). The salience of both sad mood and QOL for pacemaker implantation patients is highlighted by their status as strong psychosocial predictors for heart disease (82) and independent correlates of physical comorbidities and increased mortality risk (83). “Appetite” was another symptom directly associated with QOL in the network analysis. Poor appetite is common symptom among older adults as well as those who have pacemaker implantations (84). Among the potentially relevant appetite changes, digestive problems, sense perception impairments (e.g., loss of taste, smell and/or appetite) and chewing or swallowing difficulties can affect eating and/or food intake, contribute to weight loss and lead to nutritional deficiencies related to lower QOL (83).

Although strengths of this research included its focus on depression in an understudied population based on both traditional analysis and network analysis approaches, its main limitations should also be noted. First, given the highly specialized nature of the sample, the sample size was relatively small and slightly under-powered; replications are needed in future studies with large sample sizes. Second, due to a cross-sectional design, directions of causality between depression and pacemaker implantation could not be determined. Third, because the study was conducted through the National Clinical Research Center for Cardiovascular Diseases in Beijing, generalizability of findings cannot be made across other regions of China. Fourth, potential confounding influences such as the use of medications and comorbid chronic diseases were not assessed in an effort to maintain reasonable response burdens for unpaid research volunteers in this study. Such factors warrant attention in future extensions.

## 5. Conclusion

In conclusion, depression was common among patients who had undergone pacemaker implantation during the COVID-19 pandemic. Reports of a poor perceived health status, more severe anxiety symptoms and heightened fatigue were identified as unique predictors of overall depression scores. Network analysis revealed central symptoms (e.g., “Sad mood”, “Poor Energy”, and “Guilt”) and symptoms linked to QOL (e.g., “Sad mood”, “Appetite”, and “Fatigue”) that are potentially useful targets of interventions designed to prevent or reduce depression among recipients of pacemaker implantation. As suggested by the American Heart Association, clinicians should recognize potentially dynamic illness trajectories among pacemaker recipients, routinely assess patients’ psychological status, and provide timely interventions when high levels of distress are evident.

## Data availability statement

The datasets presented in this article are not readily available because the Clinical Research Ethics Committee of Beijing Anzhen Hospital that approved the study prohibits the authors from making publicly available the research dataset of clinical studies. Requests to access the datasets should be directed to Y-TX, xyutly@gmail.com.

## Ethics statement

The studies involving human participants were reviewed and approved by Beijing Anzhen Hospital. The patients/participants provided their written informed consent to participate in this study.

## Author contributions

YL, HC, and Y-TX: study design. YL, HC, H-HL, X-JS, C-YZ, and JL: data collection, analysis, and interpretation. HC, Y-LT, and Y-TX: drafting of the manuscript. TJ: critical revision of the manuscript. All authors approval of the final version for publication.
