# Supplementary material for: Prevalence of depression and its association with quality of life in patients after pacemaker implantation during the COVID-19 pandemic: A network analysis
Source: Front Psychiatry. 2023 Mar 16;14:1084792. doi: 10.3389/fpsyt.2023.1084792 (PMC10060541; doi:10.3389/fpsyt.2023.1084792)

**Supplementary materials**

Table 1. Abbreviated PHQ9 items

| items | Full name |
| --- | --- |
| PHQ1 | Anhedonia |
| PHQ2 | Sad mood |
| PHQ3 | Sleep |
| PHQ4 | Energy |
| PHQ5 | Appetite |
| PHQ6 | Guilt |
| PHQ7 | Concentration |
| PHQ8 | Motor disturbance |
| PHQ9 | Suicide ideation |

**Supplementray legend**

Figure S1. Nonparametric bootstrapped difference test

Figure S1. Nonparametric bootstrapped difference test

A:


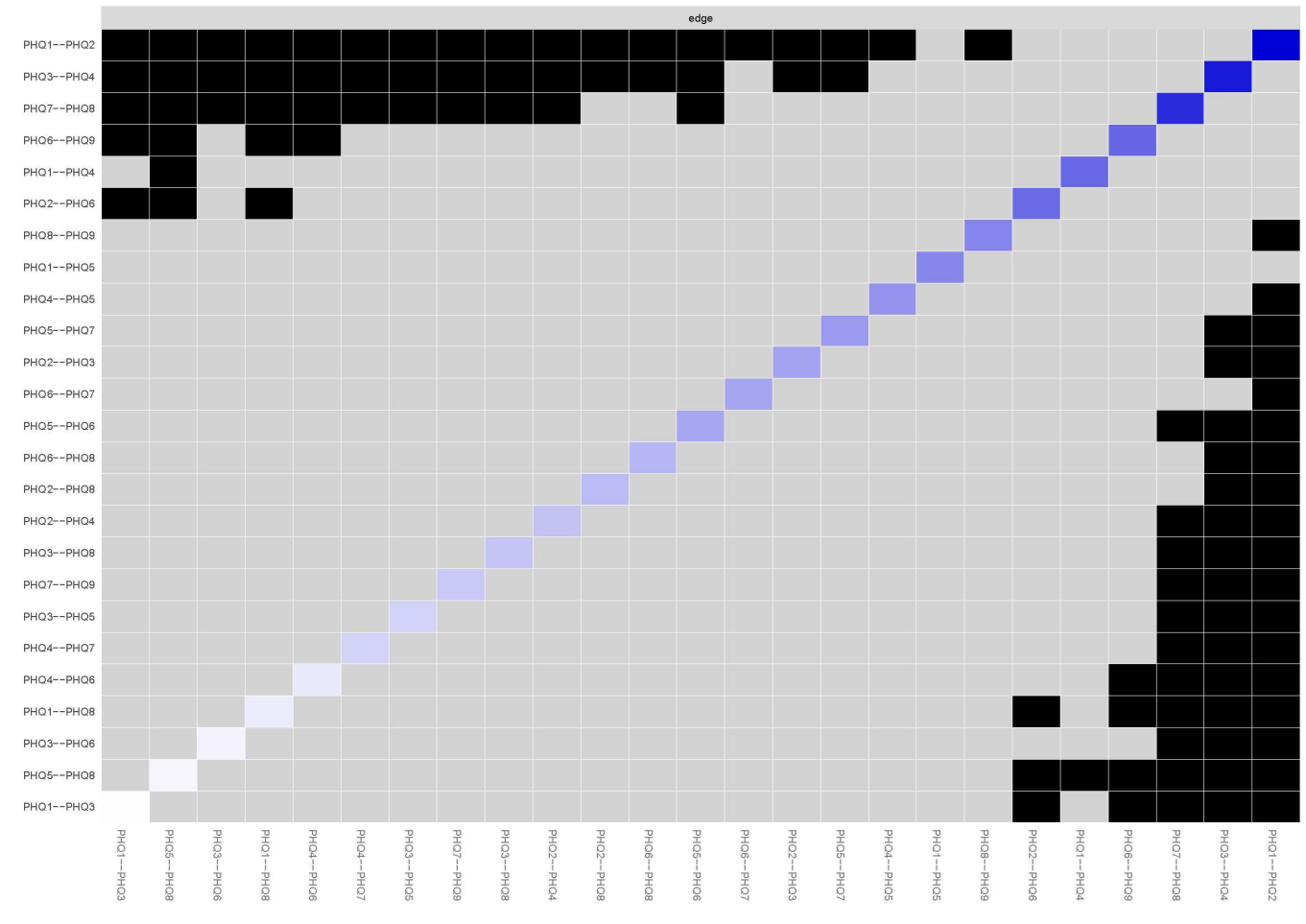


B:


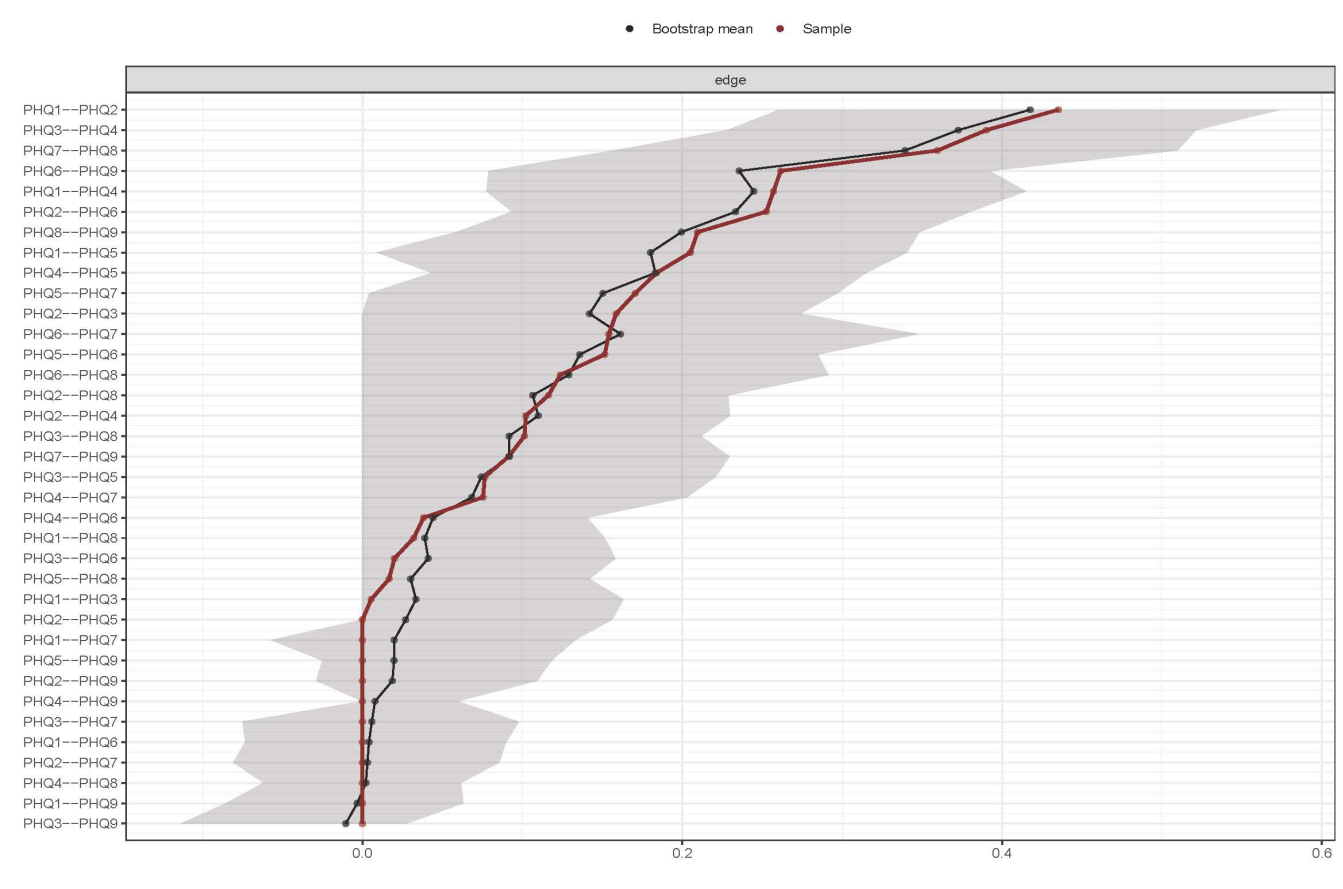

Supplement: Supplementary file 1 [file Data_Sheet_1.docx]
